# Successful Utilization of Mechanical Thrombectomy in a Presentation of Pediatric Acute Ischemic Stroke

**DOI:** 10.1155/2018/5378247

**Published:** 2018-04-15

**Authors:** Esther S. Kim, Erica K. Mason, Andrew Koons, Shawn M. Quinn, Robert L. Williams

**Affiliations:** ^1^Department of Emergency Medicine, Lehigh Valley Health Network/USF MCOM, CC & I-78, Allentown, PA 18103, USA; ^2^Diagnostic Radiology, Section of Neuroradiology, Lehigh Valley Health Network/USF MCOM, CC & I-78, Allentown, PA 18103, USA

## Abstract

Guidelines regarding the management of acute ischemic stroke (AIS) in the pediatric population using mechanical recanalization procedures are lacking. We present a case of a 14-year-old male diagnosed in the Emergency Department with an acute onset stroke who underwent successful mechanical clot removal by interventional radiology.

## 1. Introduction

Guidelines regarding the management of acute ischemic stroke (AIS) in the pediatric population are described [1–3], but the guidelines do not yet take into account the mechanical recanalization procedures [4]. More frequently, cases that describe successful endovascular revascularization in pediatric AIS have begun to show up in the literature [5–9]. We present a case of a 14-year-old male with acute onset stroke who underwent successful mechanical clot removal by interventional radiology.

## 2. Case Report

A 14-year-old male with a past medical history significant for a concussion and exercise-induced asthma presented to the Emergency Department (ED) via an ambulance after having sudden onset left-sided hemiparesis during basketball practice.

Prior to this, the patient's mother had reported that he had been complaining of headaches, neck pain, and intermittent palpitations. He had been using ibuprofen with some relief of the symptoms.

In the department, the patient was noted to have flaccid left-sided paralysis, with a left-sided facial droop (forehead sparing), and mild dysarthria. He was complaining of a 5/10 headache and nausea. The patient was otherwise alert and oriented.

**Figure 1:**
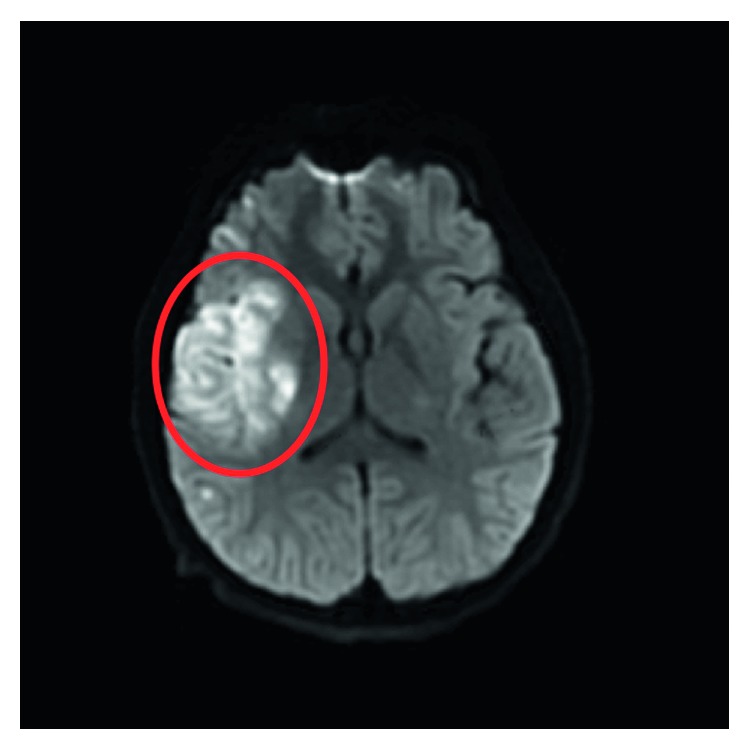
Diffusion weighted imaging (DWI) sequence demonstrating restricted diffusion in the right MCA territory of the basal ganglia and temporal lobe.

Within thirty minutes of neurological focal loss and shortly after arriving in the department, the patient's symptoms improved significantly. He was noted to have 4/5 strength in his left upper and lower extremities, with a mild sensory deficit and pronator drift of the left upper and lower extremities. He was seen by a pediatric neurologist at that time and had an NIHSS of 2. Two hours after initial resolution, while having the braces in his mouth removed, he had complete return of his previous symptoms. The patient was sent to MRI at that time which was suboptimal but showed diminished caliber of the right internal carotid artery (R ICA) and diminished flow through the right middle cerebral artery (R MCA) (Figure 1).

**Figure 2:**
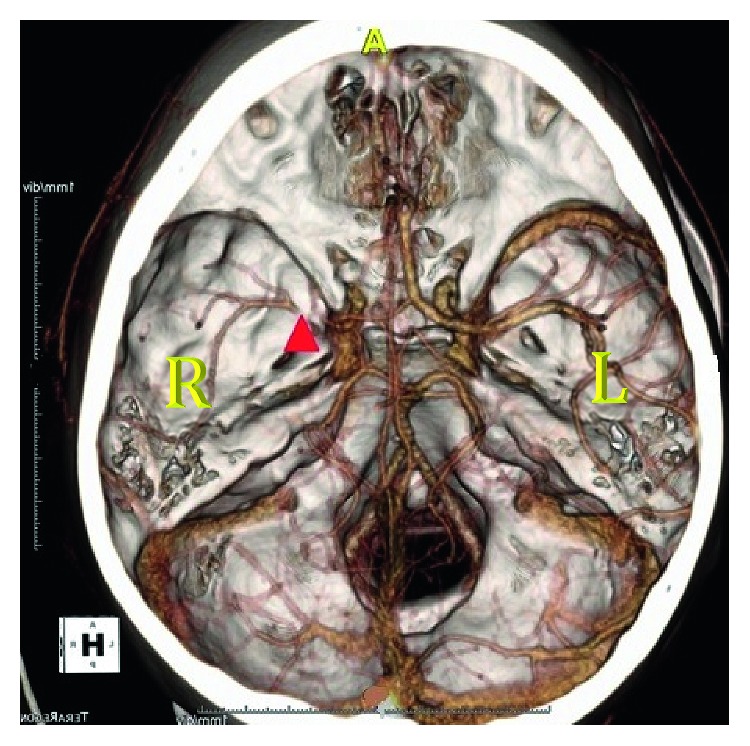
3D reconstruction of the CTA showing stenosis or embolus in the right MCA.

**Figure 3:**
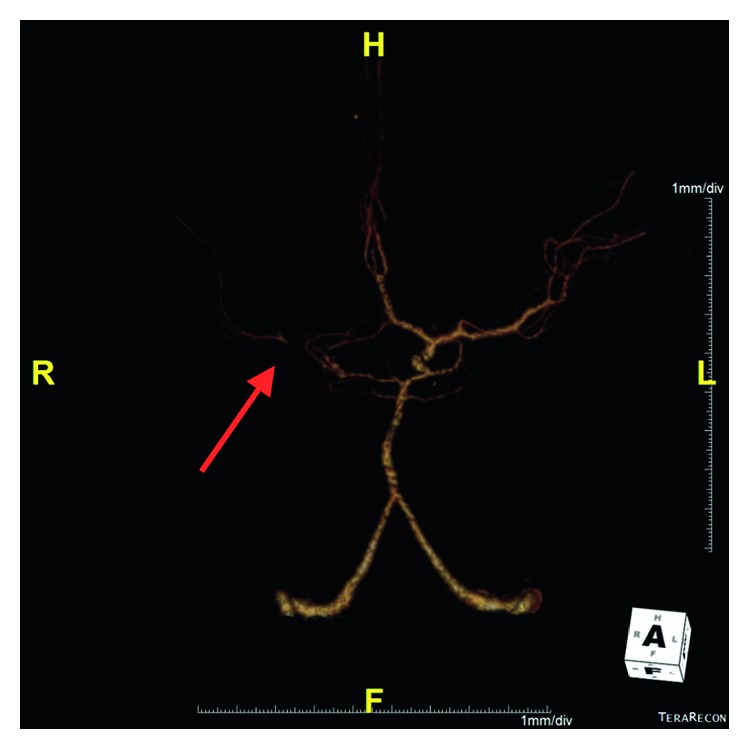
3D reconstruction of the CTA showing poor filling and blood flow in the right MCA.

**Figure 4:**
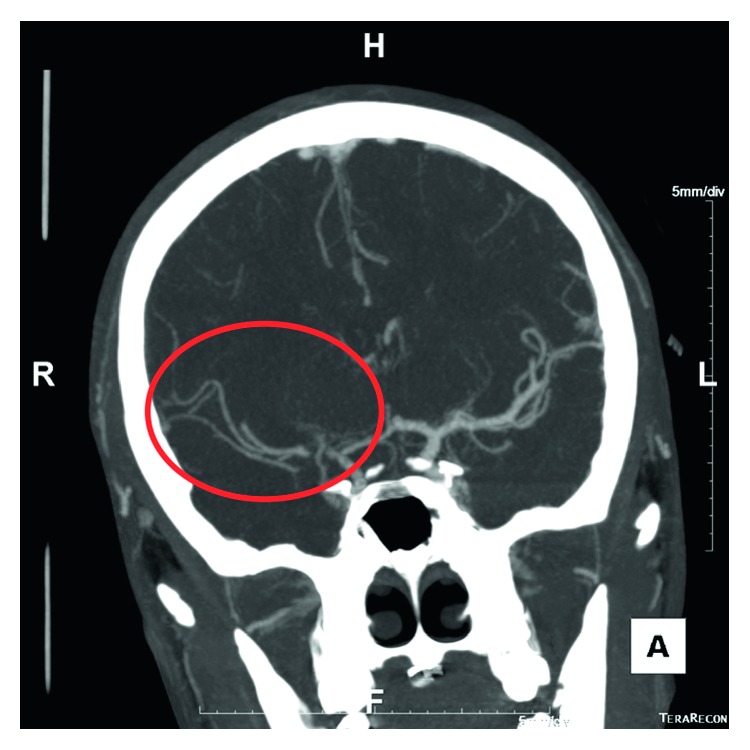
Postthrombectomy, CTA maximum intensity projection (MIP) shows improved blood flow and distal filling of the right MCA.

The patient returned to the department and was noted to be disoriented. Shortly after, he had a generalized seizure that lasted two minutes and resolved after lorazepam administration. He was intubated, CT angiogram was performed at approximately 6 hours after waxing and waning symptoms and showed an R MCA occlusion (Figures 2 and 3), and interventional radiology was contacted at this time. Arterial puncture was performed at seven hours and thirty-seven minutes from initial presentation to the Emergency Department. Interventional neuroradiology was able to perform a mechanical thrombectomy of the R ICA and R MCA with successful clot retrieval (Figure 4). Specifically, eight hours and 24 minute after arrival, a second pass with a Trevo retriever 4 × 30 mm (Stryker) with intermediate catheter aspiration was performed with a Catalyst 6 (Stryker). Base catheter was a FlowGate (Stryker).

The patient was extubated the following day. At that time, he had 4/5 strength in the left upper and lower extremities and a mild facial droop. After discharge, the patient participated in intensive physical therapy and occupational therapy with significant improvement in symptoms. At short-term follow-up, the patient had returned to school and was running track. Serial follow-up MRIs were obtained. At over 3 months postevent, brain MRI showed sequela of prior multifocal infarcts in the right cerebral hemisphere conforming to the R MCA vascular territory, with extensive multifocal encephalomalacia and gliosis. At over 6 months postevent, MRI showed stable sequela of multifocal infarcts in the R MCA territory. No new lesions within the brain were seen. Clinically, at over 6 months postevent, his only physical deficit from the AIS was decreased fine movement dexterity of his left fingers.

## 3. Discussion

Pediatric emergency medicine providers encounter AIS infrequently, and it is therefore not uncommon for considerable delay to occur in the diagnosis [7]. While the pediatric mortality is described to be between 5 and 10% and is favorable when compared to adult patients, pediatric survivors of AIS are left with persistent neurologic and psychosocial deficits or seizure disorder for the rest of their lives [10, 11]. The relationship between seizures and stroke was documented as early as 1864; it has been more recently reported that seizures were 18 times more likely in children than adults within 24 hours of noted stroke symptoms [12, 13]. It is important for physicians to recognize this relationship not only in preparation for the likelihood of a seizure in a pediatric stroke patient but also for earlier recognition of the patient who may be seizing with an underlying stroke [14]. The cause of the stroke in pediatric patients may be embolic (cardiac etiology), from a carotid dissection, or cryptogenic. In our case, the cause of the stroke was likely intracranial arteriopathy although the thrombectomy did suggest a component of an acute clot. Until further guidelines and recommendations evolve, mechanical thrombectomy may be considered in carefully selected pediatric patients after consultation with the appropriate neurological and radiological interventional specialists [6, 15].
